# Pluripotent stem cell marker deficiency in salivary mucoepidermoid carcinoma with relevance to molecular profiling: An experimental study

**DOI:** 10.1007/s44445-025-00018-x

**Published:** 2025-06-06

**Authors:** Ebtissam Alerraqi, Abdulkarim Hasan, Essam Mandour

**Affiliations:** 1 New Giza University, Giza, Egypt; 2 Ministry of Health, Giza, Egypt; 3 Pathology Department, Faculty of Medicine, Al-Azhar University, Cairo, Egypt; 4 Pathology Department, School of Medicine, Badr University in Cairo (BUC), Cairo, 11829 Egypt

**Keywords:** Mucoepidermoid carcinoma, Cancer stem cells, Nanog, SOX2, OCT4, And MENA

## Abstract

**Aim:**

Salivary gland mucoepidermoid carcinoma (MEC) is a morphologically challenging tumor, harboring a canonical *CRTC1/3:MAML2* fusion, if investigated. However, the large cohorts available did not invesitgate the diagnosed cases adequately; leaving any mucin-producing malignancy possible MECs although >50% of salivary gland tumors secret mucin luminally or extra-luminally. This study examined the expression of stem cell markers Nanog, SOX2, OCT4, and MENA in salivary MEC using immunohistochemistry and to confer, whether or not, they may have a potential role in defining the tumoral molecular profile.

**Materials and methods:**

Forty well-investigated parotid MEC cases (p63+, p40+, CK7+, Ck5/6+, AE1/AE3+, EMA+, S100 -, ATF1 -, WT1-, SOX9 - and SOX10 -), all with *MAML2* rearrangements and without *EWSR1 *alteration, were interrogated using immunohistochemical techniques to detect the immunoreactivity for Nanog, SOX2, OCT4, and MENA. Additionally, the *POU5F1* FISH probe was used to confirm the immunohistochemical findings for OCT4.

**Results:**

Immunohistochemical analysis revealed negative or nonspecific immunoreactivity of NANOG, SOX2, and OCT4 antibodies throughout all examined specimens, inferring deficient pluripotency factor within MEC cellular oncogenesis. However, MENA was widely expressed in all cases. The results of the *POU5 F1* FISH probe were consistent with the immunohistochemical data, showing no detectable expression of OCT4, Nanog or SOX2, across all 40 samples.

**Conclusion:**

Cancer stem cells likely do not play any significant role in the pathogenesis of salivary MEC. The widespread expression of MENA, however, suggests that it has functions beyond promoting stemness or pluripotency in these tumors.

**Supplementary Information:**

The online version contains supplementary material available at 10.1007/s44445-025-00018-x.

## Introduction

Mucoepidermoid carcinoma (MEC) could be the most common malignant salivary gland tumor, if all previously reported cases were truly MECs, typically affecting the parotid gland and presenting as a painless swelling in relatively older population. Pathologically, it consists of mucous, squamous, and intermediate cells and is graded as low, intermediate, or high, with prognosis worsening as grade increases. *CRTC1/3::MAML2* fusion is present in about 75–80% of cases and aids in diagnosis. Prognosis varies, with low-grade MEC having a 90–98% survival rate, while high-grade MEC has 30–54%, with higher recurrence and metastasis risk (Adams et al. 2015; Sadeghi et al. 2022).

Detecting cancer stem cells (CSC) in mucoepidermoid carcinoma (MEC) has yielded conflicting results from various methodic approaches (Adams et al. 2015; Sadeghi et al. 2022; Silva et al. 2023). The claim that neoplastic cells originate from tissue-specific stem cells in solid tumors is not supported by unequivocal evidence and is based on in-vitro studies and immunodeficient mouse models (Adams et al. 2015).

The definitive role of stem cells as cancer origins, particularly in molecularly labeled carcinomas, remains debatable. In MEC, the CSC's oncogenic role is poorly understood due to a lack of suitable research models (such as cell lines and xenograft models (Zhang et al. 2014)) and human molecular clones that can identify tumorigenic cell subpopulations (Zhang et al. 2014).

The robustness of CSC studies is also hampered by the complex spatial heterogeneity of intratumoral maximal stemness or pluripotency, potentially confounded by overlapping molecular signatures with epithelial-mesenchymal transition (EMT) and tumor invasion processes (Schoenhals et al. 2009; Han et al. 2014; Ramachandran et al. 2014). The heterogeneous distribution of stemness-related transcription factors such as sex-determining region Y-box 2 (SOX2), octamer-binding transcription factor 4 (OCT4), and Nanog homeobox (NANOG), within the tumor microenvironment can lead to sampling bias, affecting the accuracy of CSC quantification. Moreover, stemness markers such as cluster of CD44 and ALDH1 are also implicated in EMT and invasion, which complicates their specificity as definitive CSC markers. CD44 contributes to EMT by interacting with activating SNAIL and TWIST1, leading to E-cadherin suppression and cytoskeletal reorganization. These overlapping roles make it challenging to distinguish whether observed changes in stemness-related gene expression reflect true CSC functionality or are secondary to EMT and invasive tumor progression (Engels et al. 2012; Kong et al. 2010; Weygant et al. 2015).

CSC markers frequently studied in carcinomatous lesions include OCT4 (Schoenhals et al. 2009; Ramachandran et al. 2014; Lim et al. 2011; Featherston et al. 2016), SOX2 (Vijayakumar et al. 2020; Thankappan et al. 2022), Nanog (Xu et al. 2017; Virant-Klun et al. 2016; Tsai et al. 2011; Ezeh et al. 2005), and Mammalian‐enabled (MENA) (Hu et al. 2017). To investigate whether MEC might originate from committed progenitor cells, not true stem cells or intrinsic stemness could be a secondary driver of MEC progression, this study assigns molecular assay of the *POU5F1* gene alongside immunohistochemical analysis of its protein product, OCT4, and other co-expressed proteins (MENA, SOX2, and Nanog). *POU5F1* encodes OCT4, a key factor maintaining pluripotency. OCT4, SOX2, and Nanog form a core network that activates stem cell genes and suppresses differentiation. MENA, an actin-associated protein, is not directly regulated by these factors but may be influenced during cell motility and EMT (Han et al. 2014; Ramachandran et al. 2014).

## Material and methods

### Cases and grading

The study was approved by the University Ethical Review Board. A G-power analysis was conducted with an alpha (α) level of 0.05, a power of 90%, and an effect size of 0.731. This analysis predicted that a sample size of 40 cases would be needed. Forty archived formalin-fixed, paraffin-embedded blocks of MEC and ten normal salivary tissue samples were initially enrolled. The included cases were graded according to the Armed Forces Institute of Pathology (AFIP) grading system (Xie et al. 2020). Low-grade MECs are well-circumscribed, predominantly cystic, and rich in mucous cells, with minimal nuclear atypia and low mitotic activity. Intermediate-grade MECs exhibit a balanced mix of mucous, epidermoid, and intermediate cells, with both cystic and solid areas and moderate nuclear atypia. High-grade MECs are primarily solid, composed mainly of epidermoid and intermediate cells, showing marked pleomorphism, high mitotic activity, necrosis, and perineural invasion.

Clinicopathological data were obtained from private pathology laboratories and Dr EM's registry, including patient age, sex, tumor site, histological features, and clinical outcomes. Clinical outcomes were classified based on patient survival and disease progression. Inclusion criteria encompassed enrolling parotid mucoepidermoid carcinoma specimens that demonstrated positive immunoreactivity for squamous differentiation markers p63 and p40, cytokeratin 7, cytokeratins 5/6, OSCAR, pan-cytokeratin AE1/AE3 establishing epithelial origin, and epithelial membrane antigen (EMA) supporting glandular differentiation, while concurrently exhibiting negative immunostaining for S100 protein, activating transcription factor 1 (ATF1), PanTRk, GATA3, DOG1, Wilms tumor 1 (WT1) , and sex-determining region Y-box transcription factors SOX9 and SOX10. All cases demonstrated confirmed MAML2 gene rearrangements through fluorescence in situ hybridization (FISH) analysis, representing the canonical molecular hallmark of mucoepidermoid carcinoma, while simultaneously lacking EWSR1 gene alterations to exclude hyalinizing clear cell carcinoma and other EWSR1-rearranged clear-cell salivary malignancies. A'good' or 'favorable'outcome was defined as the absence of disease recurrence or metastasis during follow-up, while a 'poor' outcome was characterized by tumor recurrence, metastasis, or disease-related mortality. The exclusion criteria included cases without a confirmed MEC diagnosis upon re-evaluation, cases with unavailable paraffin blocks, and patients with incomplete clinical data.

### Immunohistochemical staining

Tissue samples (4 µm) were deparaffinized, rehydrated, and antigen-retrieved using Tris–EDTA buffer (pH 8.0) at 97 °C for 30 min. After cooling, the slides were washed in phosphate-buffered saline (PBS) to remove excess buffer. Endogenous peroxidase activity was blocked by incubating the slides in 3% hydrogen peroxide for 10 min. Non-specific binding sites were blocked by incubating the sections with 5% normal goat serum for 30 min at room temperature. Following primary antibody incubation, the slides were washed in PBS and incubated with an appropriate biotinylated secondary antibody (1:200, Vector Laboratories) for 1 h at room temperature. The reaction was visualized using the Vectastain Elite ABC Kit (Vector Laboratories), with 3,3'-diaminobenzidine (DAB) as the chromogen. Counterstaining was performed with hematoxylin for 1 min to visualize the tissue architecture. Slides were then dehydrated, cleared in xylene, and mounted with a coverslip. Positive and negative controls were included for each IHC run to validate the staining procedure.

The primary antibodies used included anti-Nanog (dilution of 1:100, recombinant anti-Nanog antibody, Abcam), anti-OCT-4 (SC-8629; 1:50 dilution; Santa Cruz Biotechnology), anti-SOX2 (Monoclonal, MA5-1389, ThermoFisher), and anti-MENA (monoclonal, clone dilution 1:500, Dako, Agilent Technologies, Santa Clara, CA) antibodies, with hematoxylin counterstaining and background reduction steps.

Positive controls were validated on appropriate tissues using an Olympus CX41 microscope (magnification 40 ×). Seminoma was used as a control for Nanog and OCT4 (nuclear staining), Squamous cell carcinoma for SOX2 (nuclear) and breast carcinoma for MENA (nuclear and membranous). IHC staining intensity of anti-MENA is graded on a 0 to 3 + scale: 0 (Negative): No staining as follows, 1 + (Weak): 1–10% positive cells, 2 + (Moderate): 10–70% Clearly visible staining but not intense, 3 + (Strong): > 70% Dark, intense staining.

### Molecular testing

For fluorescence in situ hybridization (FISH) analysis on FFPE sections, a *POU5F1* dual-color break-apart probe (Empire Genomics, USA) was purchased to process the samples. Cells with *POU5F1* gene rearrangements should exhibit separated orange and green signals not in close vicinity on all filters. The *POU5F1* dual-color break-apart probe was used in FISH to detect structural alterations or rearrangements in the *POU5F1* gene at a cut-off value of 10%. For accuracy, tissue samples from a confirmed salivary clear-cell myoepithelial carcinoma with *POU5F1* alterations were used as positive controls and normal salivary tissue samples were used as negative controls. Both controls were processed simultaneously for validation. All studied cases were positive for *MAML2*.

### Statistical calculations

Other than measuring descriptive statistics, immunohistochemical expression levels (Nanog, SOX2, OCT4, and MENA) were compared to survival outcomes and histologic grading was assessed through log-rank and Cox proportional hazards regression analyses using Statistical Package for Social Sciences 26.0 (IBM, Chicago). Log-rank and Cox proportional hazards regression analyses were used to analyze the association between immunohistochemical expression levels and survival outcomes using the Kaplan–Meier method.

## Results

### Clinicopathological findings

MEC slightly favors females (22 patient; 55%) over males (18 patient; 45%), affecting individuals across a broad age range (12–62 years, mean 42.4 ± 12.5), predominantly as low-grade MEC (LG-MEC) subtypes (Supplementary Table 1).

The distribution of diagnoses shows the prevalence of LG-MEC, constituting the majority at 21 (52.5%). Intermediate grade MEC (IG-MEC) represents 15 (37.5%) of the cases, while high-grade MEC (HG-MEC) accounts for 4 (10%) of cases. This breakdown emphasizes the spectrum of aggressiveness within MEC subtypes, providing critical information for prognosis and treatment planning. The clinical TNM staging provides valuable insights into the extent of tumor spread in the studied cases. The majority of cases (28 cases; 70%) exhibit a localized tumor without regional LN involvement or distant metastasis (cT2 N0M0). However, variations in staging, including cases with LN involvement and distant metastasis, underscore the clinical heterogeneity within the cohort. The variability in age and sex distribution highlighted the wide-ranging impact of MEC across the same population. Most cases shared a common clinical TNM stage of cT2 N0M0. The clinical prognosis was retrieved from the follow-up archives. Cases 16, 27 and 29 showed poor outcome (death of disease) although the corresponding histologic grading was LG-MEC. Moreover, Case 21, which demonstrated advanced stage (cT2 N2M1) and classified as high-grade but showed no adverse outcomes. Table 1 shows the grading criteria relating to the 40 patients of parotid MEC enrolled in the study.

**Table 1:** Histologic profile of the 40 parotid cases and their grading according to the AFIP system

Case	cTNM	Cysts	PNI	Necrosis	Mitoses/10 HPF	Anaplasia	AFIP grading system
1	cT2 N0M0	15	Yes	Yes	4	Yes	**Low**
2	cT2 N0M0	40	No	No	3	No	**IG**
3	cT2 N0M0	25	Yes	Yes	3	No	**IG**
4	cT2 N1M0	20	Yes	Yes	3	No	**IG**
5	cT1 N2M1	35	Yes	Yes	3	No	**High**
6	cT2 N0M0	20	Yes	No	3	Yes	**Low**
7	cT2 N0M0	40	Yes	No	2	No	**Low**
8	cT2 N0M0	45	No	No	1	No	**IG**
9	cT2 N0M0	40	No	No	3	No	**IG**
10	cT2 N0M0	20	Yes	No	3	Yes	**IG**
11	cT2 N0M0	30	No	Yes	1	No	**Low**
12	cT2 N0M0	20	No	No	2	No	**IG**
13	cT2 N1M0	25	Yes	Yes	3	No	**Low**
14	cT2 N1M0	35	No	No	2	No	**Low**
15	cT2 N0M0	30	No	No	1	No	**Low**
16	cT2 N0M0	20	No	No	2	No	**Low**
17	cT2 N0M0	45	Yes	Yes	3	No	**IG**
18	cT2 N0M0	40	Yes	No	2	No	**Low**
19	cT2 N0M0	30	No	No	1	No	**Low**
20	cT2 N0M0	20	Yes	Yes	3	No	**IG**
21	cT2 N2M1	15	Yes	Yes	4	No	**High**
22	cT2 N0M0	35	No	No	2	No	**Low**
23	cT2 N2M1	40	Yes	No	2	No	**High**
24	cT2 N0M0	40	No	No	3	No	**Low**
25	cT2 N0M0	25	Yes	Yes	3	No	**IG**
26	cT2 N0M0	20	No	No	2	No	**Low**
27	cT2 N1M0	30	No	No	1	No	**Low**
28	cT2 N0M0	15	Yes	Yes	4	No	**Low**
29	cT2 N1M0	30	Yes	Yes	3	No	**Low**
30	cT2 N0M0	40	No	No	3	No	**IG**
31	cT2 N0M0	20	Yes	Yes	4	Yes	**Low**
32	cT2 N0M0	20	Yes	Yes	3	No	**Low**
33	cT3 N1M0	35	No	No	2	No	**IG**
34	cT3 N2M1	45	Yes	Yes	3	No	**High**
35	cT2 N0M0	30	No	Yes	1	No	**Low**
36	cT2 N0M0	25	Yes	Yes	3	No	**IG**
37	cT2 N1M0	20	Yes	No	3	Yes	**Low**
38	cT2 N1M0	20	Yes	No	3	No	**IG**
39	cT2 N0M0	40	Yes	No	3	Yes	**Low**
40	cT2 N0M0	35	No	No	2	No	**IG**

IG: Intermediate grade; LVI: Lymphovascular involvement; PNI: perineural invasion; 10 HPF: 10 high power fields; AFIP grading system: Armed Forces Institute of Pathology grading system

### Immunohistochemical findings

**Fig. 1:**
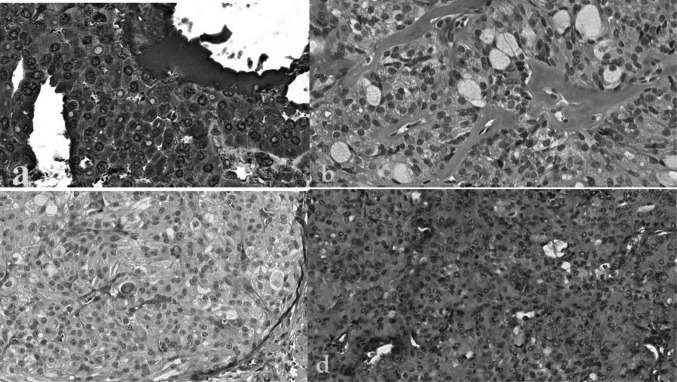
Photomicrographs showing **a**) Positive nuclear and cytoplasmic immunostaining for MENA in conventional LG- MEC (400x); **b**) Negative immunostaining for Nanog in oncocytic LG-MEC (400x); **c**) Negative immunostaining for SOX2 in oncocytic LG-MEC (200x); **d**) Negative immunostaining for OCT4 in solid HG-MEC (100 × original magnification)

The current study showed nuclear and cytoplasmic immunohistochemical overexpression of MENA in all MEC cases, irrespective of grading and staging. Nevertheless, all normal salivary glands tested negative for the anti-MENA antibody. The 40 cases showed negative immunostaining for Nanog, SOX2, and OCT4 (Fig. 1). It should be noted that statistical analysis was not informative.

### Molecular findings

**Fig. 2:**
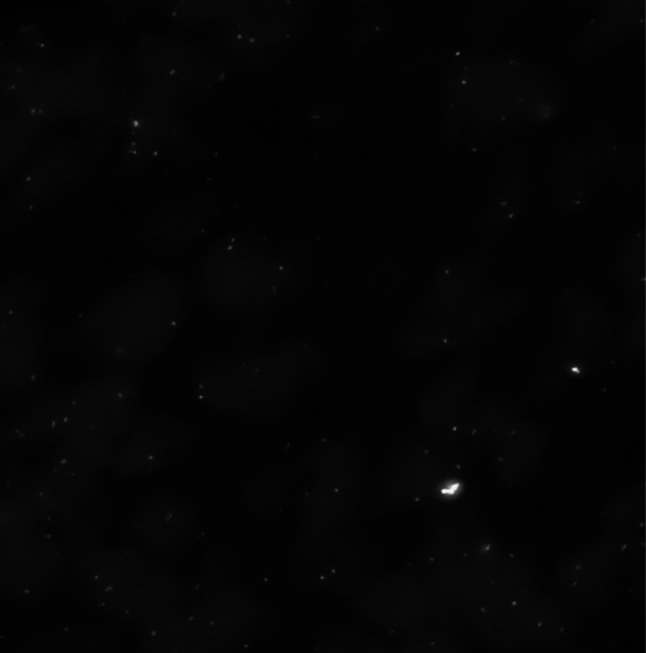
Photomicrograph showing retained signals for *POU5F1*

Molecular findings revealed no POU5F1 alterations using the dual-color break-apart probe (Fig. 2). In this context, a dual-color break-apart probe is typically employed in FISH to identify structural alterations or rearrangements in a specific gene, in this case, *POU5F1*.

### Statistical analysis findings

**Fig. 3:**
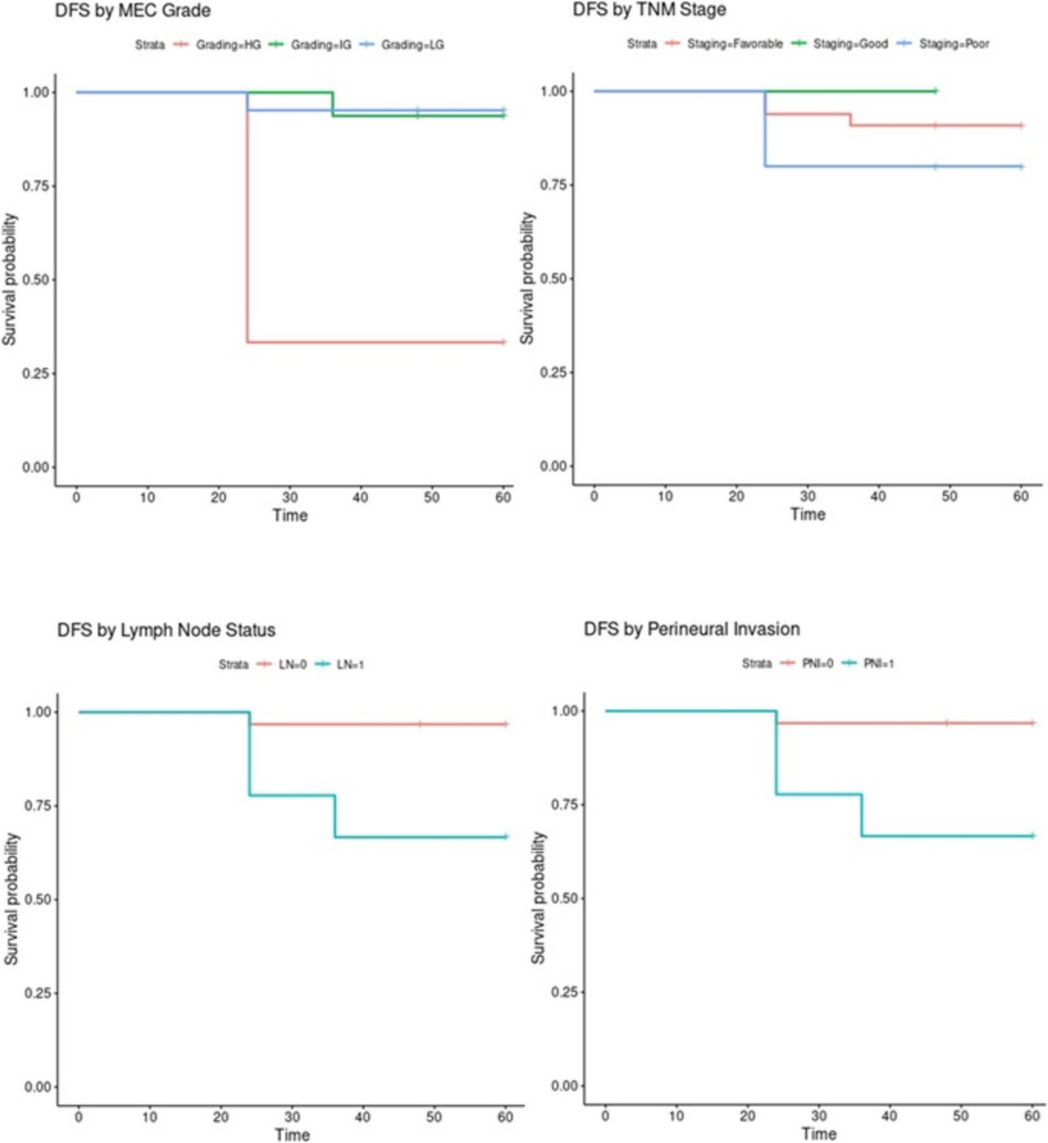
Kaplan–Meier survival plots

Kaplan–Meier survival plots demonstrated a significant reduction in 5-year Disease-Free Survival (DFS) in high-grade cases, those with nodal metastasis, and advanced TNM clinical stages (III + IV) (Fig. 3). Table 2 shows the Chi-square calculations and corresponding p-values.

**Table 2:** Chi-square calculations for LN, TNM, grading and staging

Variable	N	(O-E)^2/E	(O-E)^2/V	Chi-sq	df	p-value
Grading	40	5.1429	6.000	6.3	39	0.04
Staging	40	5.143	6.000	6.0	39	0.01
LN	40	1.55	6.98	7.0	39	0.008
PNI	40	1.55	6.98	7.0	39	0.008

Immunohistochemical analysis revealed consistent expression patterns across all cases, with all markers either consistently positive or consistently negative. This lack of variability precluded meaningful comparisons, and as a result, Cox regression analysis did not identify significant differences between the markers.

## Discussion

Experimental research was conducted on salivary gland neoplasms to explore the microenvironmental alterations (Fekry et al. 2020; Abelmeguid et al. 2022; Ibrahim et al. 2022; Ahmad 2019; Elshorkoubally et al. 2020; Li Mow Chee et al. 2023). Analyzing CSC solely based on the expression of specific immunohistochemical markers is an oversimplification that introduces potential confounding factors by co-targeting irrelevant expressions (Lim et al. 2011; Featherston et al. 2016; Vijayakumar et al. 2020; Thankappan et al. 2022). This approach also overlooks subsets of CSC with distinct marker profiles, preventing accurate attributions to specific pathways.

In our study, the MENA expression in all MEC cases was diffusely positive (cytoplasmic and membranous), regardless of the grade. However, the observed nuclear expression in MEC did not reach a threshold considered to be positive staining when compared to the non-specific background. This finding aligns with previous reports, where benign salivary neoplasms, including 10 pleomorphic adenomas and 10 Warthin's tumors, exhibited negative immunostaining for MENA, whereas positive expression was observed in a few cases of salivary duct carcinomas, carcinomas ex pleomorphic adenoma, acinic cell carcinomas, squamous cell carcinomas, and high-grade MEC (Gurzu et al. 2012). Given this pattern, post-translational modifications (e.g., phosphorylation) of cytoplasmic MENA might be more critical, altering its interaction with actin cytoskeletons and modulating integrin signaling or focal adhesion kinase activity. A recent study on oral carcinomas indicates that dysregulated MENA phosphorylation affects cytoskeletal dynamics and cell adhesion, highlighting its potential as a prognostic biomarker and therapeutic target in oral squamous cell carcinoma (Na et al. 2022).

None of the MEC cases showed cytoplasmic or nuclear immunoreactivity for SOX2, Nanog, or OCT4. This lack of expression, observed both in normal salivary gland tissue and neoplastic samples for other stem cell markers, suggests alternative mechanisms of tumor progression that do not rely on these classical stem cell markers. It is possible that other, less-characterized markers may be involved in driving the cancer’s aggressive features. Unlike previous studies on Nanog, SOX2, OCT4 in salivary carcinomas (Ramachandran et al. 2014; Xu et al. 2017; Nanduri et al. 2013; Li et al. 2014) and *POU5F1* (Oliveira Moura et al. 2021; Möller et al. 2008; Antonescu et al. 2011), the consistent immunonegativity observed in this study, along with the absence of molecular alterations in *POU5 F1 *in *MAML2*-rearranged MECs, indicates that POU5F1 pseudogenes (e.g., POU5F1B) or splice variants (e.g., POU5 F1- δA) do not contribute to MEC progression. This lack of detection further suggests that stemness in MEC may be regulated through alternative pathways not dependent on these markers.

These observations lead to mechanistic considerations. The dysregulated phosphorylation of emerin in MECMECs could disrupt normal cellular functions and contribute to tumorigenic signaling pathways by modulating the accessibility of oncogenes and tumor suppressors. While direct studies on emerin phosphorylation in mucoepidermoid carcinoma (MEC) are limited, research in other cancers suggests a significant role for emerin in tumor progression (Li Mow Chee et al. 2023; Liddane and Holaska 2021).

Perturbations in nuclear architecture (e.g., altered lamin A/C expression) and chromatin repositioning could modulate the accessibility of oncogenes (e.g., *CCND1*) or tumor suppressors (e.g., *TP53*, *RB1*) in oncogenesis. Dysregulated chromatin organization, potentially through aberrant histone modifications (H3K27me3, H3K4me3) or DNA methylation changes at CpG islands, may contribute to the dysregulation of key genes governing cell cycle (e.g., CDKN1A, CDK4), differentiation, and apoptosis (e.g., BCL2, BAX)22. Although regulatory aberrations may upregulate cytoplasmic MENA levels (via miR-21 inhibition of MENA's 3'UTR), altered membrane localization (through palmitoylation or CAAX motif changes) could affect integrin-mediated adhesion (α5β1, αvβ3) or EGFR/FAK signaling. However, nuclear MENA expression, indicative of transcriptional modulation, is posited as the primary driver of pathogenic gene expression changes (e.g., upregulating VIM, MMP9) in HG-MEC cases.

Given that stem-marker-based investigation of MEC (e.g., exosome analysis, whole transcriptome assay, and methylation profiling) is understudied, or molecularly unverified cases were tested for hyper/hypomethylation, benchmarking results on MECs is not possible (Adams et al. 2015; Marleen et al. 2022). Future studies with broader molecular profiling approaches are necessary to further elucidate the role of these markers in MEC pathogenesis. Future research may emphasize the regulation of MENA localization, CSC suppression and cross-talk; interaction with non-canonical fusion and epigenetic maintenance of stemness.

## Conclusion

​ Without contributing to the dispute over the MECs versus Non-MECs, we demonstrated expression of a few CSC markers in 40 *MAML2*-rearranged MECs. MENA’s ability to translocate across the nuclear envelope in MEC into the cellular cytoplasm suggests a transcriptional modulation, yet its immunohistochemical non-specific expression does not encode oncogenic signaling patterns. Additionally, the lack of expression of pluripotency antibodies (Nanog, SOX2, and OCT4) coupled with *POU5*F1's normal molecular arrangement disputes the cancer stemness paradigm in MEC. However, epigenetic modifications, rather than genetic changes, might regulate *POU5*F1/OCT4 in MEC. Additionally, other stemness factors with different subcellular localizations (e.g., *MENA11a, MENA∆6*, *KLF4*) may play a more significant role in MEC pathogenesis.

To date, the following questions remain unanswered: Does nuclear MENA initiate transformation, with cytoplasmic effects being downstream? What is the potential for aberrant cytoplasmic MENA signaling to drive nuclear translocation, the capacity of MEC microenvironments to suppress canonical CSC marker expression while maintaining cryptic stemness through *CRTC1/3::MAML2*-collaborative non-coding RNA networks, and the function of bivalent chromatin domains in sustaining poised pluripotency states without active transcription factor expression. Given that DNA methylation profiling clusters MEC with hyalinizing clear cell carcinoma (HCCC), shared CpG island hypermethylation patterns may silence pluripotency genes in both malignancies, while MENA potentially regulates DNA methyltransferase activity contributing to epigenome remodeling and histone deacetylase complexes may interact with MENA to establish repressive chromatin architecture. The salivary-HCCC methylome convergence raises questions about common developmental origins versus convergent epigenetic evolution during carcinogenesis, and the therapeutic potential of demethylating agents to reactivate dormant stemness programs in MEC warrants investigation.

## Supplementary Information

Below is the link to the electronic supplementary material.

Supplementary file1 (DOCX 21 KB)

## Data Availability

Available upon request from the corresponding author.
