# Supplementary material for: Pluripotent stem cell marker deficiency in salivary mucoepidermoid carcinoma with relevance to molecular profiling: An experimental study
Source: Saudi Dent J. 2025 Jun 6;37(4-6):16. doi: 10.1007/s44445-025-00018-x (PMC12143996; doi:10.1007/s44445-025-00018-x)
Supplement: Supplementary file 1 — Supplementary file1 (DOCX 21 KB) [file 44445_2025_18_MOESM1_ESM.docx]

**Table 1**. Clinicopathological profile of the 40 parotid cases.

| **Case** | **Age/sex** | **AFIP Grading** | **cTNM** | **SOX2** | **Nanog** | **OCT4** | **MENA** | **LNI** | **rPNI** | **Clinical outcome** |
| --- | --- | --- | --- | --- | --- | --- | --- | --- | --- | --- |
| 1 | 44/M | LG-MEC | cT2N0M0 | - | - | - | +++ | No | No | Favorable |
| 2 | 49/F | IG-MEC | cT2N0M0 | - | - | - | +++ | No | No | Good |
| 3 | 52/M | IG-MEC | cT2N0M0 | - | - | - | +++ | No | No | Favorable |
| 4 | 57/F | IG-MEC | cT2N1M0 | - | - | - | +++ | Yes | Yes | Favorable |
| 5 | 42/M | HG-MEC | cT1N2M1 | - | - | - | +++ | Yes | Yes | Poor |
| 6 | 40/F | LG-MEC | cT2N0M0 | - | - | - | +++ | No | No | Favorable |
| 7 | 42/M | LG-MEC | cT2N0M0 | - | - | - | +++ | No | No | Favorable |
| 8 | 34/M | IG-MEC | cT2N0M0 | - | - | - | +++ | No | No | Good |
| 9 | 40/F | IG-MEC | cT2N0M0 | - | - | - | +++ | No | No | Favorable |
| 10 | 43/M | IG-MEC | cT2N0M0 | - | - | - | +++ | No | No | Favorable |
| 11 | 43/F | LG-MEC | cT2N0M0 | - | - | - | +++ | No | No | Favorable |
| 12 | 56/F | IG-MEC | cT2N0M0 | - | - | - | +++ | No | No | Favorable |
| 13 | 45/M | LG-MEC | cT2N1M0 | - | - | - | +++ | Yes | Yes | Favorable |
| 14 | 54/F | LG-MEC | cT2N1M0 | - | - | - | +++ | Yes | Yes | Favorable |
| 15 | 62/F | LG-MEC | cT2N0M0 | - | - | - | +++ | No | No | Favorable |
| 16 | 57/F | LG-MEC | cT2N0M0 | - | - | - | +++ | No | No | Poor |
| 17 | 42/M | IG-MEC | cT2N0M0 | - | - | - | +++ | No | No | Favorable |
| 18 | 39/F | LG-MEC | cT2N0M0 | - | - | - | +++ | No | No | Favorable |
| 19 | 12/M | LG-MEC | cT2N0M0 | - | - | - | +++ | No | No | Favorable |
| 20 | 34/M | IG-MEC | cT2N0M0 | - | - | - | +++ | No | No | Favorable |
| 21 | 19/M | HG-MEC | cT2N2M1 | - | - | - | +++ | Yes | Yes | Favorable |
| 22 | 34/F | LG-MEC | cT2N0M0 | - | - | - | +++ | No | No | Favorable |
| 23 | 41/F | HG-MEC | cT2N2M1 | - | - | - | +++ | Yes | Yes | Poor |
| 24 | 47/M | LG-MEC | cT2N0M0 | - | - | - | +++ | No | No | Favorable |
| 25 | 28/F | IG-MEC | cT2N0M0 | - | - | - | +++ | No | No | Favorable |
| 26 | 36/M | LG-MEC | cT2N0M0 | - | - | - | +++ | No | No | Favorable |
| 27 | 50/F | LG-MEC | cT2N1M0 | - | - | - | +++ | Yes | Yes | Poor |
| 28 | 39/M | LG-MEC | cT2N0M0 | - | - | - | +++ | No | No | Favorable |
| 29 | 46/F | LG-MEC | cT2N1M0 | - | - | - | +++ | Yes | Yes | Poor |
| 30 | 31/M | IG-MEC | cT2N0M0 | - | - | - | +++ | No | No | Favorable |
| 31 | 58/F | LG-MEC | cT2N0M0 | - | - | - | +++ | No | No | Favorable |
| 32 | 42/F | LG-MEC | cT2N0M0 | - | - | - | +++ | No | No | Favorable |
| 33 | 40/F | IG-MEC | cT3N1M0 | - | - | - | +++ | Yes | Yes | Poor |
| 34 | 42/M | HG-MEC | cT3N2M1 | - | - | - | +++ | Yes | Yes | Poor |
| 35 | 34/F | LG-MEC | cT2N0M0 | - | - | - | +++ | No | No | Favorable |
| 36 | 40/F | IG-MEC | cT2N0M0 | - | - | - | +++ | No | No | Favorable |
| 37 | 43/F | LG-MEC | cT2N1M0 | - | - | - | +++ | Yes | Yes | Favorable |
| 38 | 43/M | IG-MEC | cT2N1M0 | - | - | - | +++ | Yes | Yes | Favorable |
| 39 | 56/F | LG-MEC | cT2N0M0 | - | - | - | +++ | No | No | Favorable |
| 40 | 45/M | IG-MEC | cT2N0M0 | - | - | - | +++ | No | No | Favorable |

**Abbreviations**: F: Female; HG-MEC: High-grade MEC; IG-MEC: Intermediate grade MEC; LG-MEC: Low-grade MEC; LN: Lymph node involvement; M: Male; rPNI: Radiographic perineural invasion.
